# Multivalent Exosome Based Protein Vaccine: A “Mix and Match” Approach to Epidemic Viruses’ Challenges

**DOI:** 10.3390/vaccines13030258

**Published:** 2025-02-28

**Authors:** Mafalda Cacciottolo, Li-En Hsieh, Yujia Li, Michael J. LeClaire, Ciana L. Mora, Christy Lau, Charles Dwyer, Kristi Elliott, Minghao Sun

**Affiliations:** Capricor Therapeutics, Inc., 10865 Road to the Cure, San Diego, CA 92121, USA; mcacciottolo@capricor.com (M.C.); llhsieh@capricor.com (L.-E.H.); yli@capricor.com (Y.L.); mleclaire@capricor.com (M.J.L.); cmora@capricor.com (C.L.M.); clau@capricor.com (C.L.); cdwyer@capricor.com (C.D.); kelliott@capricor.com (K.E.)

**Keywords:** exosome, SARS-CoV-2, severe acute respiratory syndrome coronavirus 2, spike, influenza, H3, Respiratory syncytial virus, RSV, lentiviral system, COVID-19, vaccine, therapeutic

## Abstract

**Background:** Endemic viruses are becoming increasingly the norm, and the development of a rapid and effective vaccine is necessary. **Methods:** Here, we used our StealthX^TM^ exosome platform to express either Influenza H3 (StealthX^TM^-Hemagglutinin, STX-H3), SARS-CoV-2 Delta spike (StealthX^TM^-Spike, STX-S) or respiratory syncytial virus proteins (StealthX^TM^-RSV fusion protein, STX-RSV) protein on the membrane surface and facilitate their trafficking to the exosomes. **Results:** The administration of exosomes carrying one of the antigens by themselves resulted in a strong immune response with the production of a potent humoral and cellular immune response in mice. Interestingly, these effects were obtained with the administration of nanograms of protein and without adjuvant. We tested the possibility of manufacturing a multivalent vaccine by combining exosomes expressing either STX-H3, STX-RSV or STX-S exosomes in the same formulation, in a “mix and match” approach. Mice immunized with the cocktail vaccine showed an increased immune response against all three antigens received. **Conclusions:** The results further demonstrated that our STX trivalent cocktail vaccine elicited a strong immune response, and the magnitude of the responses was comparable to the single antigen administered individually. Our data show that our exosome platform has enormous potential to revolutionize vaccinology by rapidly facilitating antigen presentation, to tackle the fast-evolving viral infections.

## 1. Introduction

The US Center for Disease Control and Prevention (CDC) has declared the SARS-CoV-2 endemic virus, a status already held by Influenza and Respiratory syncytial virus (Influenza, COVID-19, and Respiratory Syncytial Virus Vaccination Coverage Among Adults—United States, Fall 2024|MMWR). The pharmaceutical companies have come together to rapidly produce a vaccine to contrast the infections and reduce the spread, with a significant positive effect on reducing the death count and the long-lasting post-infection effects [1].

Before the COVID-19 pandemic, influenza was one of the most important and frequent viral diseases of the respiratory system. Children ≤5 years old as well as adults ≥50 years old, patients with chronic conditions or who are immunocompromised, and women who are or plan to be pregnant during the influenza season are recommended to receive an annual vaccine [2]. For both SARS-CoV-2 and influenza, due to the characteristics of the virus replication, mutation of the viral genome is frequent [3], and vaccination against new strains is needed annually, especially in cohorts with a higher risk of severe diseases [2]. The prediction for the vaccine strain is based on the epidemiological monitoring of the circulating strains, and the vaccine strains need to be updated frequently [2]. Therefore, it is necessary for updated vaccines to be able to quickly adapt to combat the ever-changing virus.

Respiratory syncytial virus (RSV) showed an increased surge after the removal of social distancing and masking (following the COVID-19 containment) [4], resulting in a substantially increased number of hospitalizations and intensive care unit (ICU) admissions. RSV is the leading causative agent of bronchiolitis, resulting in disease and death in children, older people, and immunocompromised patients [5].

With the documented surge of these endemic respiratory viruses, the CDC has advised the population to get vaccinated against all three viruses in one or multiple visits. A multivalent vaccine would be highly preferred to reduce the number of injections and lessen the burden on the health system and costs wherever efficacy against the antigens is preserved.

We have previously shown that our exosome platform StealthX^TM^ (STX) can be used to generate a novel protein-based vaccine, and it is able to elicit a potent cellular and humoral immune response against SARS-CoV-2 spike and nucleocapsid [6,7]: administration of a dual vaccine, containing both antigens, showed immune response comparable to the monovalent vaccine, suggesting no immune competition between antigens from the same virus.

In the present study, we sought to use our exosome platform to develop a trivalent vaccine to induce immune responses against SARS-CoV-2, influenza and RSV viruses. Our results showed that mice immunized with exosomes carrying SARS-CoV-2 spike protein, influenza A (H3N2) hemagglutinin, or RSV fusion protein elicited a potent cellular immune response and produced a high titer of antibodies against target immunogens. Furthermore, mice that received the trivalent formulation also showed a comparable humoral immune response toward the three antigens formulated in the vaccine. Our data showed that the exosome-based STX vaccine platform is able to elicit robust humoral and cellular immune responses against different viral antigens simultaneously with low antigen doses in the in vivo mouse model. The platform can potentially be adapted to different target antigens or mutants to combat the fast-evolving viral infections.

## 2. Methods

**Cell lines**. Human embryonic kidney 293 T cells (293T) were purchased from ATCC (CRL-3216). The 293T cells were maintained in culture using Dulbecco’s Modified Eagle Medium (DMEM), high glucose, Glutamax™ containing 10% fetal bovine serum. 293T cells were incubated at 37 °C/5% CO_2_. FreeStyle™ 293F cells (Gibco, 51-0029) were purchased from ThermoFisher (Carlsbad, CA, USA). The 293F cells were used as a parental cell line to generate SARS-CoV-2 delta spike, Influenza A virus hemagglutinin and Respiratory syncytial virus fusion protein (RSV) expressing stable cell lines: Stealth X-Spike cells (STX-S), Stealth X-H3 cells (STX-H3) and Stealth X-RSV cells (STX-RSV). The 293F, STX-S, and STX-H3 cells were maintained in a Multitron incubator (Infors HT, Annapolis Junction, MD, USA) at 37 °C, 80% humidified atmosphere with 8% CO_2_ on an orbital shaker platform rotating at 110 rpm.

**Lentiviral vectors.** Lentiviral vectors for expression of SARS-CoV-2 spike (Delta variant B.1.617.2, NCBI accession #OX014251.1), Influenza virus hemagglutinin (A/New Yotk/392/2004, NCBI accession #YP_308839) and Respiratory syncytial virus fusion protein gene (RSV, NCBI accession #MN125707.1) were designed and synthesized from Genscript (Nanjing, Jiangsu, China) together with the two packaging plasmids (pMD2.G and psPAX2). To facilitate the trafficking of SARS-CoV-2 spike, H3 and RSV to the exosomes, the proteins were linked to the N-terminal of the exosome-specific tetraspanin CD9 by a synthetic transmembrane domain and a secretion signal peptide. Lentiviral particles for transduction were generated as previously [6,7]. Briefly, lentiviral particles for transduction were generated by transfecting 293T cells with pMG.2 (Genscript), psPAX2 (Genscript) and STX-S_pLenti (Genscript) expressing spike, STX-H3_pGenlenti (Genscript) or STX-RSV_pGenlenti (Genscript) at a ratio of 5:5:1 using Lipofectamine 3000 according to the manufacturer’s instruction. Spike, H3 and RSV lentiviral particles were collected at 72 h post-transfection and used to transduce 293F parental cells to generate STX-S, STX-H3 and STX-RSV, respectively.

**Flow cytometry.** Standard flow cytometry methods were applied to measure the expression of SARS-CoV-2 spike protein, Influenza A hemagglutinin protein or Respiratory syncytial virus (RSV) on STX-S, STX-H3 or STX-RSV cell surface, respectively. In brief, 2.5 × 10^5^ STX cells were aliquoted, pelleted and resuspended in 100 µL eBioscience™ Flow Cytometry Staining Buffer (ThermoFisher, Carlsbad, CA, USA). Cells were incubated at room temperature (RT) for 30 min protected from light in the presence of an anti-spike antibody (clone 1A9, ab273433, Abcam, Waltham, MA, USA), anti-H3 (A/Perth/16/2009) (IT-003-004M18, Immune technology, New York, NY, USA) or anti-RSV (clone AM22, 01-07-0144, Cambridge Bio, Brookline, MA, USA) labeled with Alexa Fluor^®^-647 (Alexa Fluor^®^ 647 Conjugation Kit (Fast)-Lightning-Link^®^ (Abcam, ab269823) according to the manufacturer’s protocol. Following incubation, STX cells were washed with eBioscience™ Flow Cytometry Staining Buffer (ThermoFisher, cat No 00-4222-57), resuspended in PBS and analyzed on the CytoFlex S flow cytometer (Beckman Coulter, Carlsbad, CA, USA). Data were analyzed by FlowJo 2021 (Becton, Dickinson and Company; Franklin Lakes, NJ, USA.

**STX exosome production.** STX-S, STX-H3 and STX-RSV cells were cultured in FreeStyle media (ThermoFisher, 12338018) in a Multitron incubator (Infors HT) at 37 °C, 80% humidified atmosphere with 8% CO_2_ on an orbital shaker platform. Subsequently, centrifugation steps were implemented to remove cells (300× *g* × 5 min) and cell debris (3000× *g* × 15 min), while microvesicles and other extracellular vesicles larger than ~220 nm were removed by vacuum filtration (0.22 um PES filter unit). Next, exosomes were isolated using Capricor’s large-scale purification method. Briefly, the supernatant was subjected to concentrating tangential flow filtration (TFF) on an AKTA Flux s instrument (Cytiva, Marlborough, MA, USA) and then subjected to chromatography on an AKTA Avant 25 (Cytiva).

**Nanoparticle tracking analysis.** Exosome size distribution and concentration were determined using ZetaView Nanoparticle Tracking Analysis (Particle Metrix, Inning am Ammersee, Germany) according to the manufacturer’s instructions. Exosome samples were diluted in 0.1 µm filtered 1X PBS (Gibco, 10010072) to fall within the instrument’s optimal operating range.

**Jess automated Western blot.** Detection of SARS-CoV-2 spike, H3 and RSV proteins in cell lysate and exosomes used Protein Simple’s Jess capillary protein detection system. Samples were lysed in RIPA buffer (ThermoFisher Scientific, 8990) supplemented with protease/phosphatase inhibitor (ThermoFisher Scientific, A32961), quantified using the BCA assay (ThermoFisher Scientific, 23227) and run for detection. To detect the antigen of interest, the separation module 12–230 kDa was used following the manufacturer’s protocol. Briefly, 0.8 µg of sample and protein standard were run in each capillary, probed with mouse anti-Ms-RD-SARS-CoV-2 (MAB105401, 1:10 dilution, R&D Systems, Minneapolis, MN, USA), mouse anti-H3 (IT-003-004M2, 1:50 dilution, Immune-Tech, New York, NY, USA) or anti-CD9 (13174S, 1:100 dilution, Cell Signaling, Boston, MA, USA to visualize the RSV fusion protein) followed by secondary antibody provided in Jess kits (HRP substrate used neat).

**Animal studies—Mice.** Studies were conducted according to the guidelines of the Institutional Animal Care and Use Committee (IACUC protocol EB17-004-091). Mice were fed ad libitum and sterile water; housed in groups of five at 22 °C/30% humidity and light cycles of 06:00–18:00 h with standard nesting material; and allowed free movement. To examine the efficacy of STX exosomes, age-matched BALB/c mice (female, 8–10 wks old) were anesthetized using isoflurane and received bilateral intramuscular injection (50 µL per leg, total 100 µL) of either (1) PBS (2) STX-S, (3) STX-H3, (4) STX-H3+S, (5) STX-RSV, (6) STX-RSV+S, or (7) STX-H3+RSV+S exosomes, as per the different studies reported in this paper. The booster injection was performed on day 21. Mice were monitored closely for changes in health, and weight was recorded biweekly. Blood collection was performed on day 14 and day 35. A schematic of the immunization schedule was provided as Supplementary Figure S1. Blood (~50–500 µL) was collected from the submandibular vein and processed for plasma isolation after centrifugation at 2500 g for 5 min at 4 °C.

**IgG ELISA for Spike.** Mouse IgG antibody against SARS-CoV-2 spike was measured by an enzyme-linked immunosorbent assay (ELISA) using precoated ELISA plates (IEQ-CoV-S-RBD-IgG, RayBiotech, Peachtree Corners, GA, USA) according to the manufacturer’s instructions at RT. Briefly, mouse plasma samples were diluted in a sample buffer (RayBiotech) and added to antigen-coated wells in triplicates and incubated at RT for 2 h on a shaker (200 rpm). A commercially available antibody against Spike (S1N-S58, Acro Biosystems, Newark, DE, USA) was used as a positive control. Plates were washed 3 times with wash buffer (RayBiotech) and incubated for 1 h at RT with HRP-conjugated goat anti-mouse secondary antibodies (115-035-003, dilution 1:5000, Jackson ImmunoResearch, West Grove, PA, USA) or anti-rabbit (111-035-003, dilution 1:5000, Jackson ImmunoResearch) diluted in assay buffer (RayBiotech). After 3 washes, plates were developed using TMB substrate (RayBiotech). After 15 min incubation, the reaction was stopped by adding STOP solution and absorbance at 450 nm was recorded using a BioTeck Gen5 plate reader (Agilent, La Jolla, CA, USA). Endpoint titers were calculated as the dilution that emitted an optical density exceeding 4X the PBS control group.

**IgG ELISA for H3 and RSV.** Plates (Nunc MaxiSorp, Thermofisher Scientific) were coated with 10 µg/mL of H3 protein (cat# 11715-V08H, Sino Biological, Chesterbrook, PA, USA) or RSV protein (Sino Biological, cat# 11049-V08B) in coating buffer (50 mM carbonate/bicarbonate, pH 9.6) for 4 h at RT. Plates were rinsed and blocked with Teknova Assay Buffer (Teknova, cat# 2D5220) overnight. Plasma was diluted in Teknova Assay Buffer and incubated for 2 h at RT. After washing, plates were incubated for 1 h at RT with HRP-conjugated goat anti-mouse secondary antibodies (115-035-003, dilution 1:5000, Jackson ImmunoResearch). After 3 washes, plates were developed using TMB substrate (TMSK-1000-01, Surmodics, Eden Prairie, MN, USA) for 15 min, stopped with Stop Solution (acid) and absorbance values were recorded at 450 nm on a spectrophotometer (using a BioTeck Gen5 plate reader (Agilent).

**Splenocyte isolation.** Spleens were processed for single cell isolation by mechanical disruption of the spleen pouch using a syringe stopper and passage through a 0.040 mm mesh size nylon cell strainer to remove tissue debris. Erythrocytes were lysed using ammonium chloride potassium (ACK) buffer (A1049201, ThermoFisher), and splenocytes were collected using centrifugation at 300× *g* for 5 min. The cellular pellet was resuspended in completed RPMI 1640 media (FG1215, Millipore Sigma Aldrich, Rockville, MD, USA).

**ELISPOT.** Briefly, splenocytes were isolated by mechanical disruption of the spleen pouch and seeded at a concentration of 5 × 10^5^ cells/well in a precoated 96-well plate and incubated for 24 h in the presence or absence of either 10 µg/mL of SARS-CoV-2 Spike (S1N-C52H4, AcroBiosystems, Newark, DE, USA) or H3 (Sino Biological, cat# 11715-V08H). Commercially available ELISPOT plates for evaluation of IFNg (MuIFNg, Immunospot, Cellular Technology Limited, Shaker Heights, OH, USA) were used. The assay was performed according to the manufacturer’s guidelines. Plates were analyzed using the ELISPOT reader S6ENTRY (Immunospot, Cellular Technology Limited).

**Statistical analysis.** Data were analyzed using Excel and GraphPad Prism 9.1 (Boston, MA, USA) and shown as mean ± sem. One-way ANOVA with post-hoc correction for multiple comparisons.

## 3. Results

### 3.1. Characterization of STX-H3 and STX-RSV Cell Line and Exosomes

**Figure 1:**
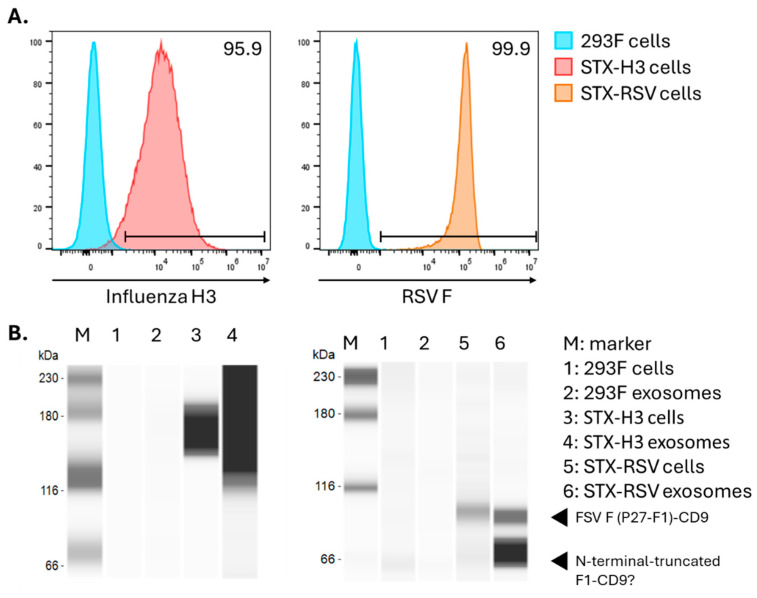
Characterization of STX-engineered cells and exosomes for the expression of influenza hemagglutinin H3 and RSV F protein. (**A**) High expression of influenza H3 protein (**left panel**, red histogram) and RSV F protein (**right panel**, orange histogram) was detected on cell surface by flow cytometry. Parent, non-engineered 293F cells (blue histograms) showed no expression of H3 and F protein, as expected. (**B**) Enrichment of influenza H3 protein (**left panel**) and RSV F protein (**right panel**) in exosomes was confirmed by Jess-automated Western Blot. From left to right: Lane M: marker (Protein Simple, Jess), Lane 1: non-engineered 293F cells, Lane 2: non-engineered 293F exosomes, Lane 3: STX-H3 cells, Lane 4: STX-H3 exosomes, Lane 5: STX-RSV cells, and Lane 6: STX-RSV exosomes. Full image of the Jess Western Blot is available as Supplementary Figure S2.

The 293F cells were engineered to express H3 or RSV fusion protein on their surface by lentiviral transduction. The expression of H3 and RSV fusion protein on the cell surface was evaluated by flow cytometry in both adherent and suspension cells. As shown in Figure 1A, 95.9% of the STX-H3 cells expressed H3 and 99.9% of STX-RSV cells expressed RSV fusion protein. STX-H3 and STX-RSV exosomes were purified according to our previously established protocol [6,7] and the purified exosomes were further characterized. Purified STX-H3 exosomes showed an average concentration of 1.63 × 10^12^ particles/mL with an expected average diameter of 141 nm and an expected polydispersity index (PDI) of 0.164. Purified STX-RSV exosomes showed an average concentration of 1.9 × 10^12^ particles/mL with an expected average diameter of 138.4 ± 1.3 nm and an expected PDI of 0.153 ± 0.009. The enrichment of H3 and RSV-F proteins was confirmed on respective exosome populations by Jess Western Blot [6,7].

### 3.2. STX-H3 Induces Strong Immune Response Against H3 Protein in Mice

The STX-H3 exosome vaccine was administered to 8–10-week-old female mice by intramuscular (IM) injection. Two IM injections were administered: a prime injection on day 1 followed by a second IM injection (referred to as boost injection) after a 3-week interval.

**Figure 2:**
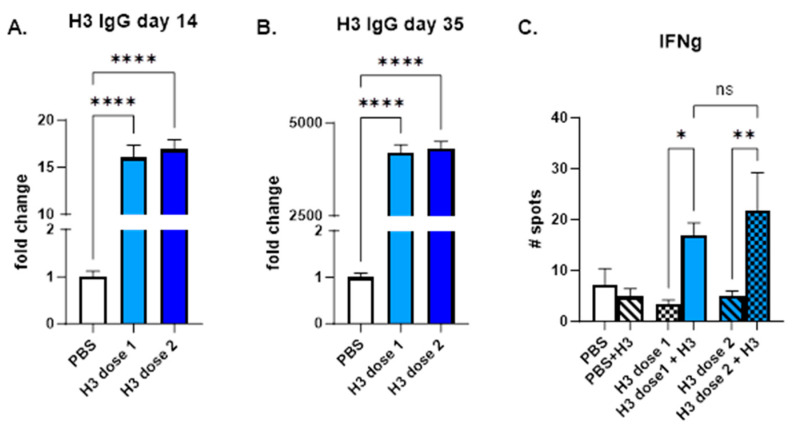
**STX-H3 exosome vaccine elicited a robust immune response.** The STX-H3 vaccine induced robust production of anti-H3 antibodies in mice after 1 (day 14) and 2 (day 35) IM injections as analyzed by ELISA (**A**,**B**). Additionally, a strong T-cell response is observed (**C**). PBS was used as a vehicle control in all studies. (**A**). STX-H3, Day 14 after a single IM injection (**B**). STX-H3, Day 35 after two IM injections. (**C**). STX-H3, IFN γ response. N = 10/experimental group. Data are shown as mean ± SEM. **** *p* < 0.0005, ** *p* < 0.01, * *p* < 0.05, ns = not significant, 1-way ANOVA.

The STX-H3 vaccine induced a strong antibody response 2 weeks after the prime injection in all animals. Mice primed with STX-H3 exosomes produced 15-fold (after one injection) to 4500-fold (after the second injection) greater anti-H3-specific IgG than control mice (Figure 2A,B). No significant difference in IgG response was observed across the two doses. To characterize the T-cell response to the STX-H3 vaccine, antigen-specific T cell responses were measured by ELISpot (Figure 2C). Splenocytes were isolated from animals at day 40 (~3 weeks after boost (2nd) injection) and evaluated using ELISpot plates precoated with interferon γ (IFNγ). PBS was used as a control in the study. Baseline expression was compared to stimulation with 10 µg/mL of H3 protein (SinoBiological). In young mice, IFNγ response was equally increased regardless of the dose used, with a 2- to 4-fold increase in IFNγ production (Figure 2C).

### 3.3. STX-RSV Induces Strong Immune Response Against RSV Protein in Mice

The quantification analysis of RSV fusion protein on STX-RSV exosomes showed that a greater amount of RSV fusion protein was delivered to the exosome membrane, resulting in increased doses for the immunization studies. A dose response study was performed using the following doses: 1. Dose 1, 1 × 10^8^ exosome/injection, 13 ng/injection; 2. Dose 2, 1 × 10^9^ exosome/injection, 132 ng/injection; 3. Dose 3, 3 × 10^9^ exosome/injection, 395 ng/injection; Dose 4, 1 × 10^10^ exosome/injection, 1316 ng/injection.

**Figure 3:**
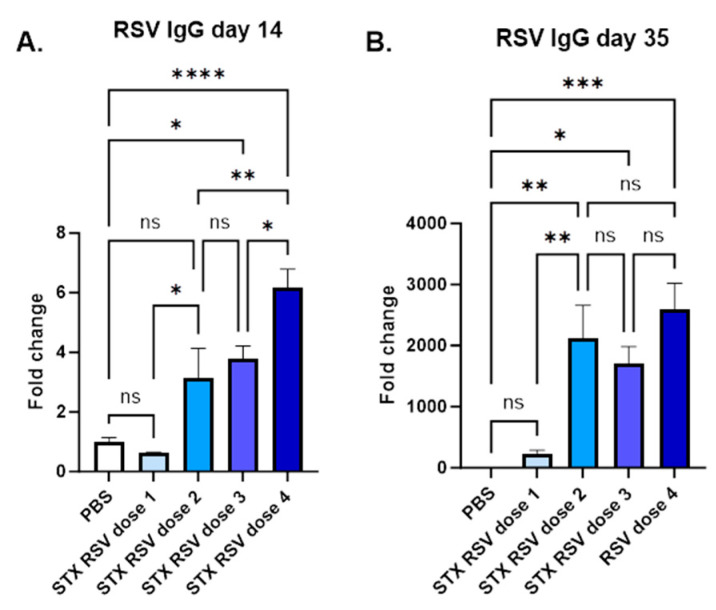
**STX-RSV exosome vaccine elicited a robust antibody response.** The STX-RSV vaccine induced robust anti-RSV antibody responses in mice after 1 (day 14) and 2 (day 35) IM injections as analyzed by IgG ELISA. PBS was used as a vehicle control in all studies. Doses: 1. Dose 1, 1 × 10^8^ exosome/injection, 13 ng/injection; 2. Dose 2, 1 × 10^9^ exosome/injection, 132 ng/injection; 3. Dose 3, 3 × 10^9^ exosome/injection, 395 ng/injection; Dose 4, 1 × 10^10^ exosome/injection, 1316 ng/injection. (**A**) STX-RSV, Day 14 after a single IM injection. (**B**) STX-RSV, Day 35 after two IM injections. N = 10/experimental group. Data are shown as mean ± SEM. **** *p* < 0.0005, *** *p* < 0.001, ** *p* < 0.01, * *p* < 0.05, ns = not significant, 1-way ANOVA.

As shown in Figure 3, a dose response is observed after 1 injection, with the lowest dose unable to deliver any immune response. On day 35, after full immunization (2 injections), no difference is observed among the higher doses (2 to 4), while the lowest dose fails to deliver an immune response. This data suggested that the number of particles is important to identify a functional dose. Additionally, nanograms of RSV fusion protein delivered by exosomes are sufficient to induce immunization, and a dose as low as 132 ng is responsible for a strong humoral immune response.

### 3.4. STX-H3+S Induces Strong Immune Response Against Both H3 and Spike Protein in Mice

**Figure 4:**
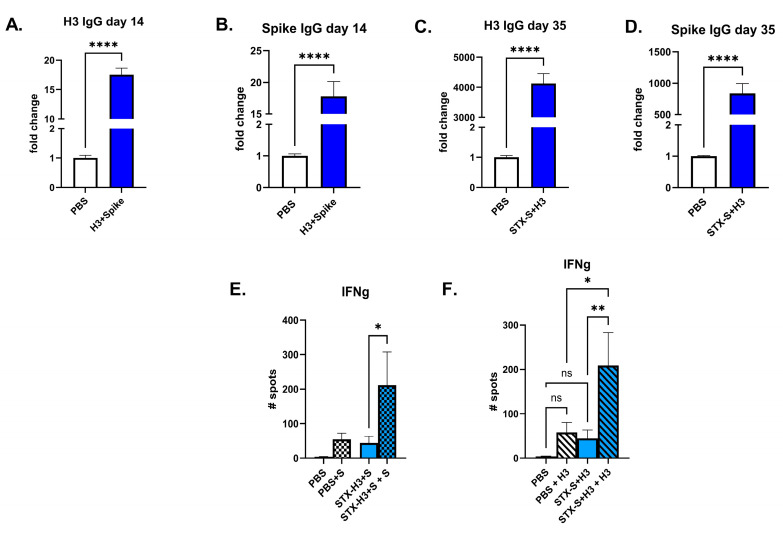
**STX-H3+S exosome vaccine elicited a robust immune response.** The STX-H3+S vaccine induced robust production of antibody against H3 and spike proteins in mice after 1 (day 14, (**A**,**B**)) and 2 (day 35, (**C**,**D**)) IM injections as analyzed by ELISA. Additionally, a strong T-cell response is observed after stimulation with either H3 or spike protein in vitro (**E**,**F**). PBS was used as a vehicle control in all studies. (**A**) Antibody response against H3 on Day 14, after a single IM injection. (**B**) Antibody response against spike on Day 14, after a single IM injection. (**C**) Antibody response against H3 on Day 35, after two IM injections. (**D**) Antibody response against spike on Day 35, after two IM injections. (**E**,**F**) IFN γ response on day 35 after H3 (**E**) or spike (**F**) stimulation. N = 15/experimental group. Data are shown as mean ± SEM. **** *p* < 0.0005, ** *p* < 0.01, * *p* < 0.05, ns = not significant, 1-way ANOVA.

We have previously shown that our STX^TM^ Technology allows us to combine STX exosomes and formulate a “cocktail” for immunization [6]. We combined STX-H3 and STX-S (expressing SARS-CoV-2 delta spike) exosomes and immunized mice by two IM injections: a first prime injection on day 1, followed by a booster injection on day 21 (3-week intervals). Antibody levels against H3 and Spike were evaluated on days 14 and 35. As shown in Figure 4A–D, all mice developed a strong immune response to both antigens. Response was comparable to previous studies using a single injection of either STX-H3 (Figure 2) or STX-S [6,7]. To characterize the T-cell response to the STX-H3+S cocktail vaccine, antigen-specific T-cell responses were measured by ELISpot (Figure 4). Splenocytes were isolated from animals at day 40 (~3 weeks after boost (2nd) injection) and evaluated using ELISpot plates precoated with IFNγ. PBS was used as a control in the study. Baseline expression was compared to stimulation with 10 µg/mL of either H3 protein (SinoBiological, Beijing, China) or Spike (AcroBiosystem). Both antigens were able to induce a 2-fold increase in IFNγ response over baseline, which did not differ from the PBS group (Figure 4E,F). This data clearly suggests that our STX platform can be used for the formulation of a multivalent vaccine without antigen competition for either the antibody or T-cell response.

### 3.5. STX-RSV+S Induces Strong Immune Response Against Both RSV and Spike Protein in Mice

**Figure 5:**
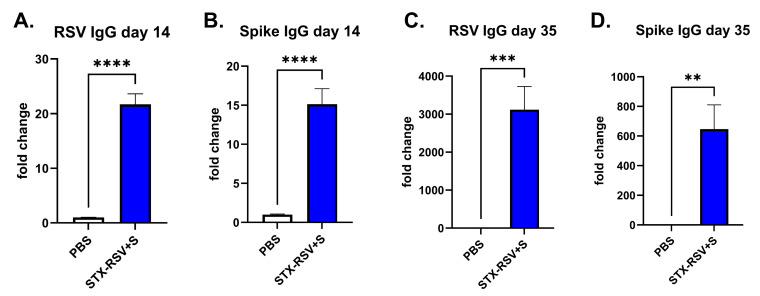
**STX-RSV+S exosome vaccine elicited a robust antibody response.** The STX-RSV+S vaccine induced robust expression of antibodies against both RSV fusion protein and SARS-CoV-2 spike antibodies in mice after 1 (day 14, (**A**,**B**) and 2 (day 35, (**C**,**D**) IM injections as analyzed by IgG ELISA. (**A**). Antibody against RSV fusion protein on Day 14, after a single IM injection (**B**). Antibody against spike on Day 14, after a single IM injection (**C**). Antibody against RSV fusion protein on Day 35, after two IM injections. (**D**). Antibody against spike on Day 35, after two IM injections. N = 10/experimental group. Data are shown as mean ± SEM. **** *p* < 0.0005, *** *p* < 0.001, ** *p* < 0.01, 2-tailed *t*-test.

We combined STX-RSV (expressing RSV fusion protein) and STX-S (expressing SARS-CoV-2 delta spike) exosomes and immunized mice with two IM injections: a first prime injection on day 1, followed by a booster injection on day 21 (3 wks interval). Antibody levels against RSV fusion protein and SARS-CoV-2 delta Spike were evaluated on days 14 and 35. As shown in Figure 5, all mice developed a strong antibody response to both antigens.

### 3.6. STX-RSV+H3+S Induces Strong Immune Response Against Both RSV Fusion Protein and Spike Protein in Mice

**Figure 6:**
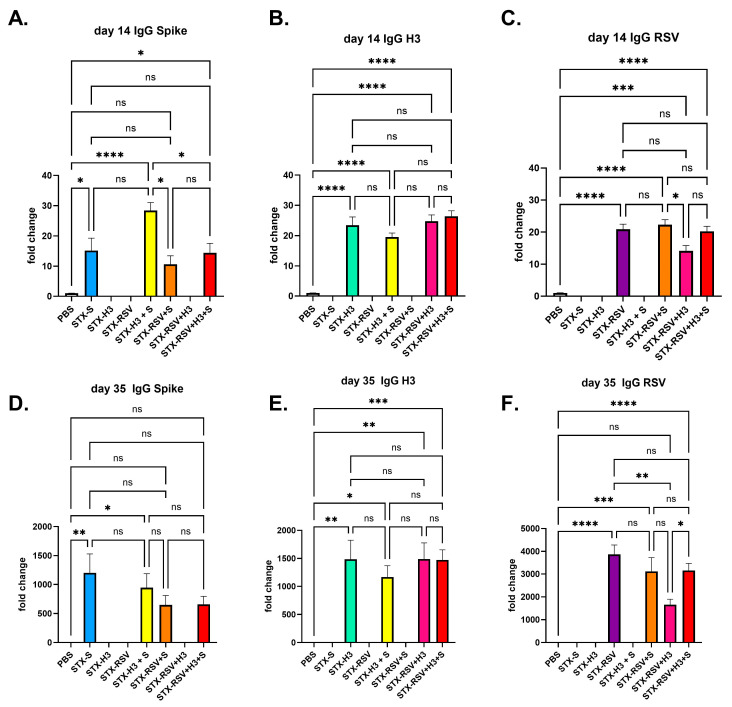
**STX-RSV+H3+S exosome vaccine elicited a robust antibody response.** The STX-RSV+H3+S vaccine induced robust humoral immunity against RSV fusion protein, Influenza H3 and SARS-CoV-2 spike in mice after 1 (day 14, (**A**–**C**) and 2 (day 35, (**D**–**F**)) IM injections as analyzed by IgG ELISA. (**A**,**D**). Antibody against Spike (**B**,**E**). Antibody against H3 (**C**,**F**). Antibody against RSV fusion protein N = 10/experimental group. Data are shown as mean ± SEM. **** *p* < 0.0005, ***p < 0.001, ** *p* < 0.01, * *p* < 0.05, ns = not significant, 1-way ANOVA.

Lastly, we tested the possibility of a multivalent vaccine. STX-S (10 ng/dose), STX-H3 (10 ng/dose) and STX-RSV (132 ng/dose) were combined in a single vaccine formulation and mice were immunized. Control group mice received either PBS, a single antigen formulation or a combination of the two. As reported in Figure 6, while no dose response study was performed to evaluate the optimal dose, when combined in a multivalent formulation, all three antigens were able to induce a strong humoral antibody response, comparable to the one obtained by single antigen immunization.

## 4. Discussion

The constant emergence of new viruses, variants of existing viruses, and the possibility of new pandemics have highlighted the need for multivalent vaccines. These multivalent vaccines must be able to strongly protect the population not only against the rapid spread of the viruses but also against their consequences on human health and society, as evidenced by the impact of the COVID-19 pandemic.

The innate characteristics of exosomes can enable them to be the next-generation vaccine: they show high bioavailability, remarkable biocompatibility, and low immunogenicity, making exosomes a very promising drug delivery candidate [8]. Because of their lipid bilayer, exosomes could potentially cross any biological barriers and deliver their cargo, endogenously or exogenously expressed [9,10,11,12,13,14,15]. Exosomes showed high stability at physiological pH and temperature, a unique advantage as a delivery system for vaccinology. Moreover, because exosomes are a natural product of any cell type, they are less likely to induce an adverse response, improving their safety profile.

We have previously shown that our StealthX^TM^ platform can easily and rapidly provide a multi-protein-based vaccine delivered by exosomes because of its easy scale-up manufacturing process, and can induce a strong immune response, demonstrated by neutralizing antibodies and a strong T-cell response with a minimum number of injections [6,7]. The platform allows multiplexing because the small amount of protein needed for exosomes carrying different antigens can be individually prepared and subsequently combined according to the desired dose and target antigens.

Here, we apply the STX technology to produce a multivalent protein-based exosome-delivered vaccine against Influenza (STX-H3), RSV (STX-RSV) and SARS-CoV-2 (STX-S). Previously, we demonstrated that STX-S and STX-N can elicit robust humoral and cellular immune responses against different variants of SARS-CoV-2 viruses [6,7]. To test the potential benefit of the trivalent vaccine, Influenza hemagglutinin H3, RSV fusion protein and SARS-CoV-2 Spike proteins were individually engineered on the exosome surface and mixed before injection. This ‘cocktail’ vaccine was able to induce a strong antibody response in mice without detectable immune competition between antigens. The immunogenicity induced by the administration of the trivalent STX-RSV+H3+S vaccine demonstrated the great potential of the StealthX^TM^ vaccine technology in controlling a broad range of infectious diseases in a single vaccine formulation.

Our data suggest that exosomes are ideal vehicles for vaccination because they can safely deliver the antigen of interest (exogenous protein) efficiently by mimicking the natural viral infection.

We are aware of the limitations of our study. A cell response was not evaluated for all vaccine combinations, and a challenge study is missing. An in vitro neutralization assay was performed previously with multi-valent vaccines manufactured using our Stealth-X platform [6,7], suggesting a neutralizing capability, regardless of the antigen used.

Many advantages have been identified for the use of the exosome-based vaccine. Because of their intrinsic property as cell-to-cell communicator effectors, exosomes can deliver vaccine components without the need for live or inactivated pathogens, reducing the risk of adverse reactions [16]. Additionally, they can be engineered to target specific cells or tissues, enhancing the precision of vaccine delivery and improving immune responses, similar to immune targeting [17]. Exosomes possess inherent immunomodulatory properties, eliminating the need for additional adjuvants, as shown here and in our previous work [6,7].

It is important to notice that because of their nature, being produced by all tissues and cells in the body, exosomes are highly biocompatible and less likely to cause adverse reactions. Moreover, they can not only carry the antigens but also protect them from degradations, ensuring an efficient delivery to the immune cells, such as dendritic cells, consequently enhancing the activation of T cells and B cells.

Vaccination is a cost-effective public health measure that can prevent disease spread and reduce disease burden. Despite the lifesaving benefits of increased vaccine uptake, vaccine hesitancy and skepticism are still longstanding barriers to the health of individuals (Centers for Disease Control and Prevention, 2019). The nanogram dose used in our formulations allows the multiplexing of several antigens: this could reduce the number of injections, with important cost-benefits, and potentially improve the vaccination rate with a broader population immunization and significant impact on health.

## 5. Conclusions

We developed a trivalent vaccine capable of simultaneously inducing immune responses (as demonstrated by the antibody levels) against multiple pathogens, including SARS-CoV-2, influenza, and RSV. The vaccination strategy can be adapted for different pathogens rapidly. With the multiplexing of different antigens, the vaccine has broader immunization capability and could be used as a booster vaccine to the existing immunity generated by previously approved vaccines. Our StealthX vaccine technology, which uses rapidly engineered and manufactured exosomes to deliver nanograms of single or multiplexed viral antigens to elicit a strong, broad immune response without any adjuvants or synthetic lipid nanoparticles, has the ability to revolutionize the next generation of vaccines.

## Data Availability

The original contributions presented in this study are included in the article/Supplementary Materials. Further inquiries can be directed to the corresponding author.

## Supplementary Materials

The following supporting information can be downloaded at: https://www.mdpi.com/article/10.3390/vaccines13030258/s1, Figure S1: Schematic diagram of in-vivo studies; Figure S2: Full images of the Jess Western Blot for Figure 1.
