# Supplementary material for: Multivalent Exosome Based Protein Vaccine: A “Mix and Match” Approach to Epidemic Viruses’ Challenges
Source: Vaccines (Basel). 2025 Feb 28;13(3):258. doi: 10.3390/vaccines13030258 (PMC11945559; doi:10.3390/vaccines13030258)
Supplement: Supplementary file 1 [file vaccines-13-00258-s001.zip › vaccines-3451134-supplementary.pdf]

Supplementary Figures

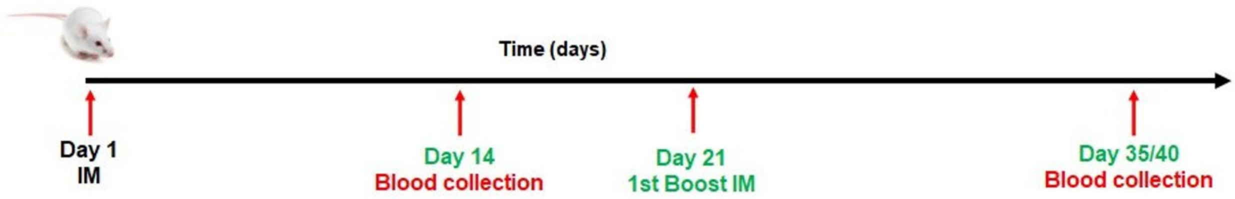

Suppl. Figure S1. Schematic diagram of in-vivo studies.

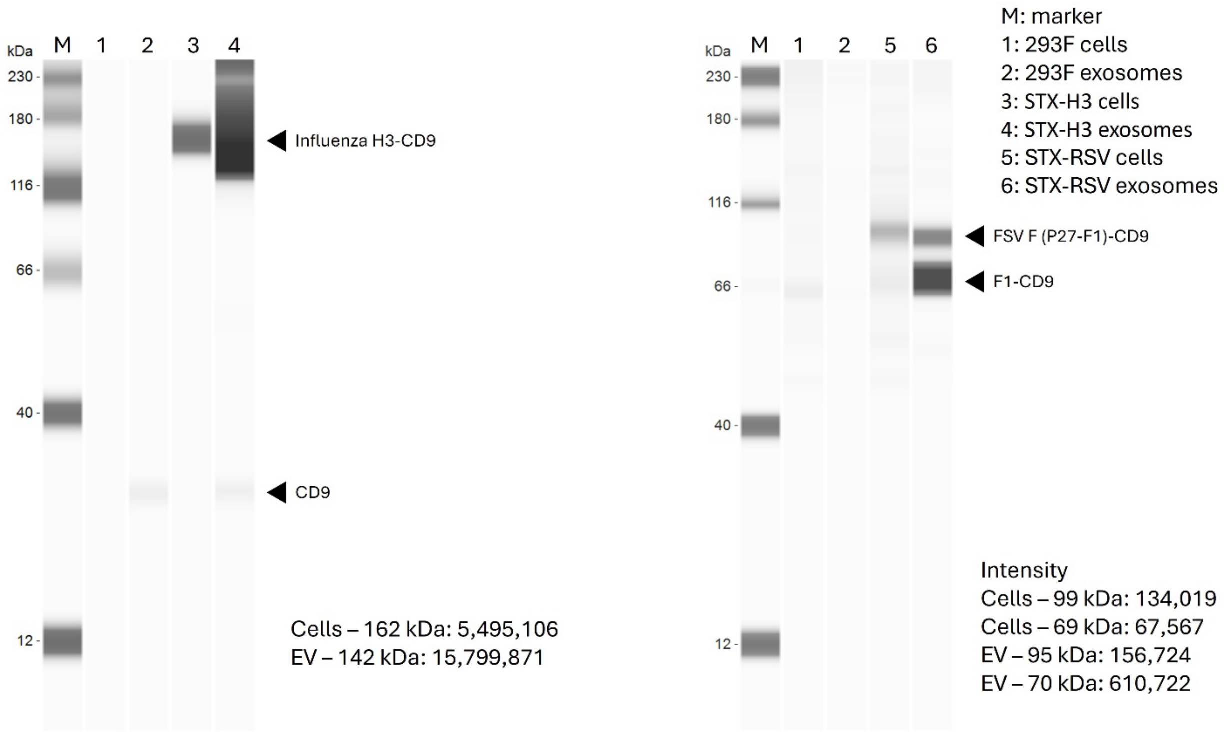

Suppl. Figure S2. Full images of the Jess Western Blot for Figure 1. Band intensity is reported for each analyzed band.
